# Polyphenols-loaded electrospun nanofibers in bone tissue engineering and regeneration

**DOI:** 10.1186/s40824-021-00229-3

**Published:** 2021-09-25

**Authors:** Iruthayapandi Selestin Raja, Desingh Raj Preeth, Mohan Vedhanayagam, Suong-Hyu Hyon, Dohyung Lim, Bongju Kim, Subramaniyam Rajalakshmi, Dong-Wook Han

**Affiliations:** 1 BIO-IT Fusion Technology Research Institute, Pusan National University, Busan, 46241 South Korea; 2 Chemical Biology and Nanobiotechnology Laboratory, AU-KBC Research Centre, Anna University, MIT Campus, Chromepet, Chennai, 600 044 India; 3 CSIR-Central Leather Research Institute, Adyar, Chennai, 600 020 India; 4 BMG Inc., Kyoto, 601-8023 Japan; 5 Department of Mechanical Engineering, Sejong University, Seoul, 05006 South Korea; 6 Dental Life Science Research Institute / Innovation Research & Support Center for Dental Science, Seoul National University Dental Hospital, Seoul, 03080 South Korea; 7 Department of Cogno-Mechatronics Engineering, College of Nanoscience & Nanotechnology, Pusan National University, Busan, 46241 South Korea

**Keywords:** Bone tissue regeneration, Electrospun nanofiber, Polyphenols, Drug loading

## Abstract

Bone is a complex structure with unique cellular and molecular process in its formation. Bone tissue regeneration is a well-organized and routine process at the cellular and molecular level in humans through the activation of biochemical pathways and protein expression. Though many forms of biomaterials have been applied for bone tissue regeneration, electrospun nanofibrous scaffolds have attracted more attention among researchers with their physicochemical properties such as tensile strength, porosity, and biocompatibility. When drugs, antibiotics, or functional nanoparticles are taken as additives to the nanofiber, its efficacy towards the application gets increased. Polyphenol is a versatile green/phytochemical small molecule playing a vital role in several biomedical applications, including bone tissue regeneration. When polyphenols are incorporated as additives to the nanofibrous scaffold, their combined properties enhance cell attachment, proliferation, and differentiation in bone tissue defect. The present review describes bone biology encompassing the composition and function of bone tissue cells and exemplifies the series of biological processes associated with bone tissue regeneration. We have highlighted the molecular mechanism of bioactive polyphenols involved in bone tissue regeneration and specified the advantage of electrospun nanofiber as a wound healing scaffold. As the polyphenols contribute to wound healing with their antioxidant and antimicrobial properties, we have compiled a list of polyphenols studied, thus far, for bone tissue regeneration along with their in vitro and in vivo experimental biological results and salient observations. Finally, we have elaborated on the importance of polyphenol-loaded electrospun nanofiber in bone tissue regeneration and discussed the possible challenges and future directions in this field.

## Background

**Fig. 1:**
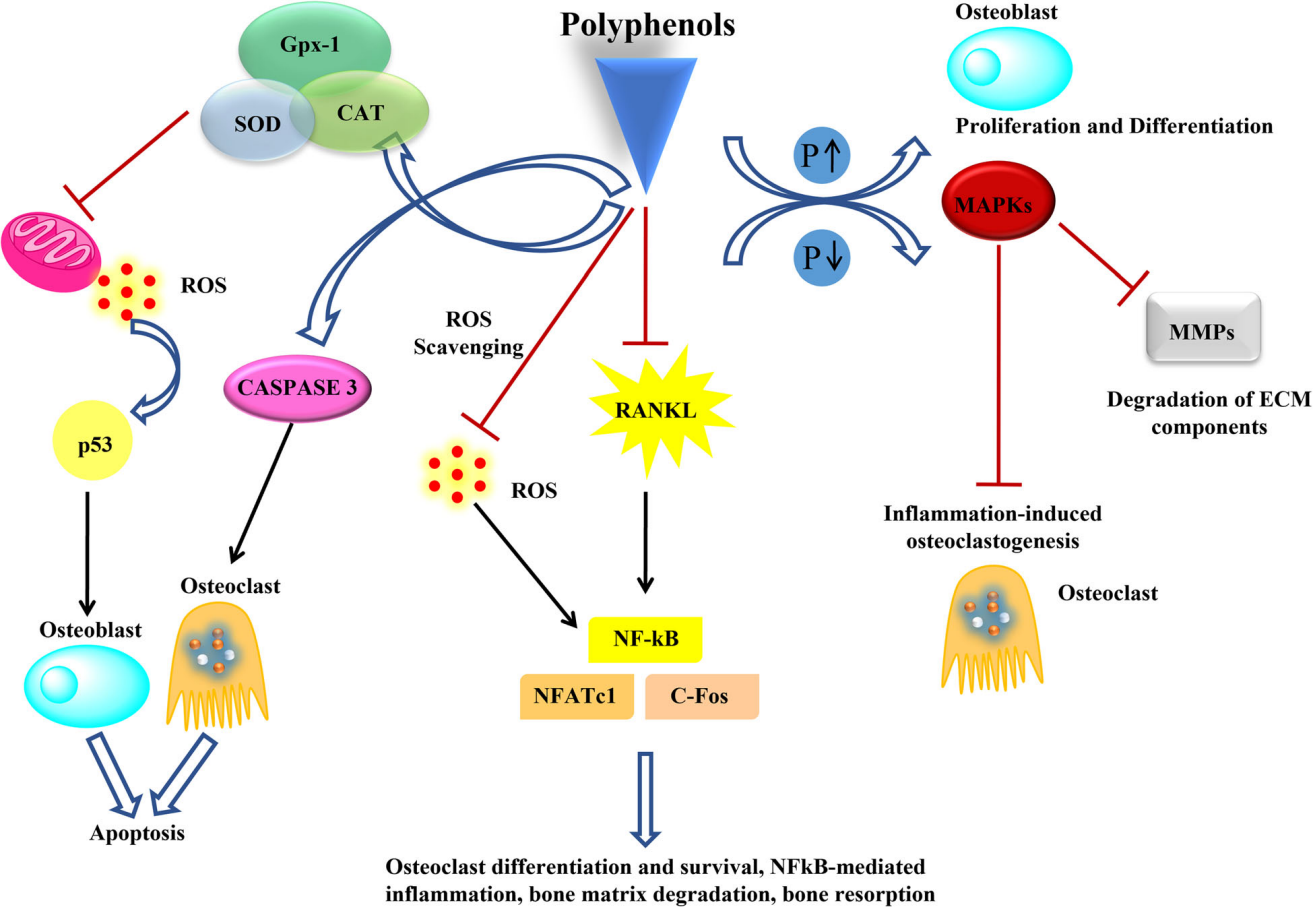
Demonstration of molecular signaling pathways of polyphenols involved in bone tissue regeneration. ROS- reactive oxygen species; p53- tumor suppressor; Gpx-1- glutathione peroxidase 1; SOD- superoxide dismutase; RANKL- receptor activator of nuclear factor kappa-Β ligand; NF-κB- nuclear factor kappa-light-chain-enhancer of activated B cells; NFATc1- nuclear factor of activated T cells 1; c-Fos- proto-oncogene; MAPKs- mitogen-activated protein kinases; MMPs- matrix metalloproteinases; ECM- extracellular matrix [13]

Plant polyphenols are excellent sources of natural antioxidants and antimicrobials, acting as potential drugs in modern biomedicine [1, 2]. Tissue regeneration and remodeling is one of the tedious and complex processes in bone tissue regeneration. Polyphenols have been promising bioactive micronutrients to safeguard and maintain bone health [3–5]. Plenty of research works have been reported to study the intriguing effects of polyphenols, likely antimicrobial, antioxidant and anti-inflammatory activity playing a vital role in bone tissue engineering [6–8]. The polyphenols’ action to maintain the balance is attributed to their hydroxyl substituents’ hydrogen bond donating ability [9]. Maintaining redox equilibrium is a critical factor in tissue engineering during the angiogenesis process, an essential step to promote long-term survival and engraftment of bone. Polyphenols can be classified into four major groups, flavonoids, lignans, stilbenes, and phenolic acids depending on the number of reactive phenolic units [10]. The flavones and catechins are the most potent flavonoids to protect the body from the reactive oxygen species (ROS) [11]. The mechanisms of polyphenols’ antioxidant action include (1) scavenging ROS, (2) up-regulation or protection of antioxidant defenses, and (3) suppression of ROS formation either by inhibition of enzymes or by chelating trace elements involved in the free radical generation [12]. Several polyphenolic compounds such as curcumin, quercetin, catechin, icariin, EGCG, and resveratrol have been studied to apply bone tissue engineering. They initiate upregulation of several biochemical pathways by scavenging free radicals and mediate the expression of inflammatory cytokines involved in bone tissue remodeling, as shown in Fig. 1 [14, 15]. Characteristic inhibition of nuclear factor kappa-Β (NF-κB), cyclooxygenase-2 (COX-2), protein-lysine 6-oxidase (LOX), and inducible nitric oxide synthase (iNOS) and activation of activating protein-1 (AP-1), mitogen-activated protein kinase (MAPK), protein kinase C (PKC), nuclear factor-erythroid 2-related factor 2 (Nrf2), and phase II antioxidant detoxifying enzymes are affiliated to the anti-inflammatory activities of the polyphenols [16]. Polyphenols demarcate the inflammatory responses, control the osteoclast’s activation process, and activate the osteoblast’s production through various signaling proteins such as RANKL, osteoprotegerin (OPG), etc. [17].

**Fig. 2:**
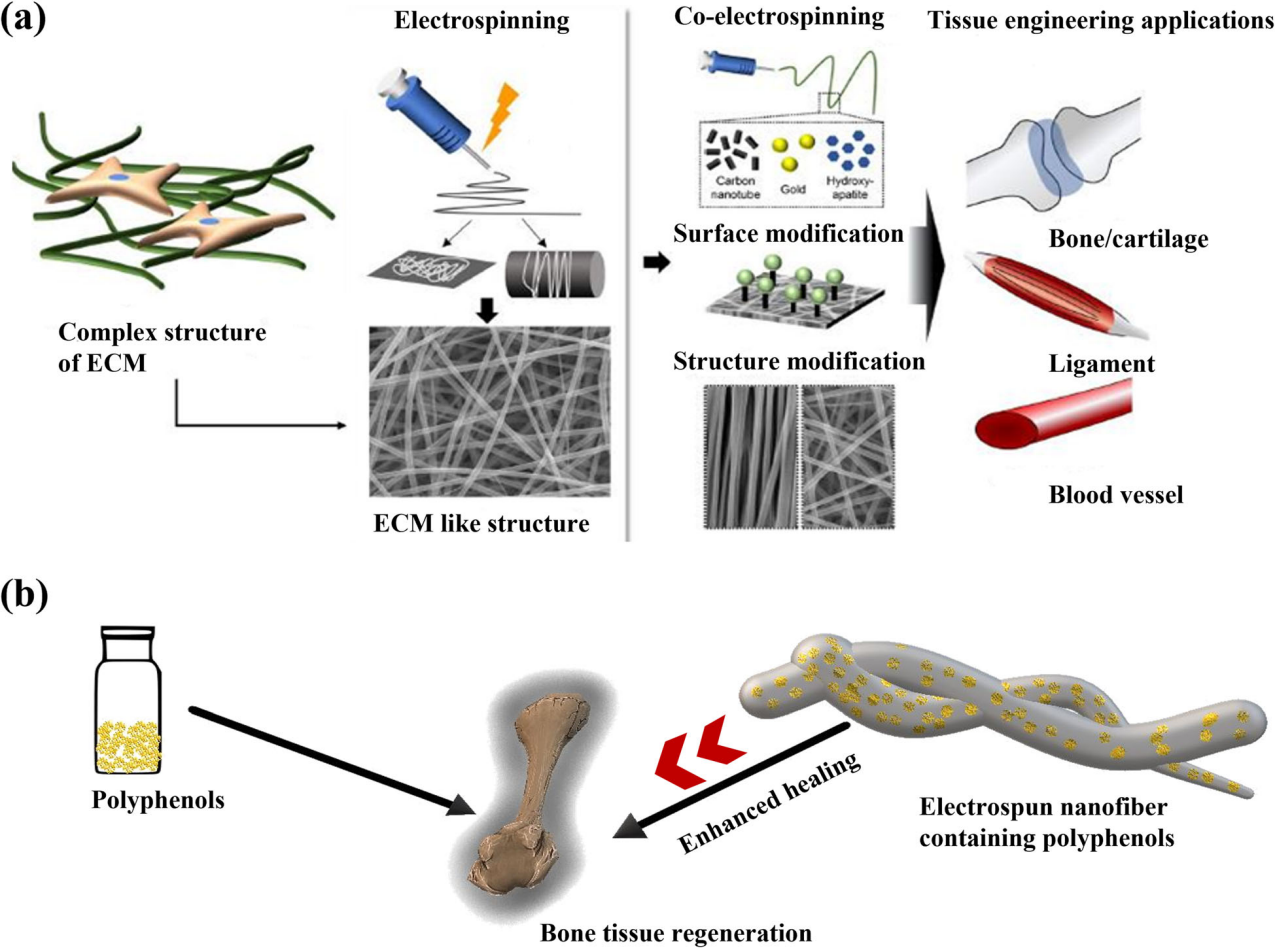
Electrospun nanofibers imitating extracellular matrix (ECM). **a** Major types of electrospinning and post-modification of electrospun nanofibers for the application of bone tissue engineering are demonstrated [31]. **b** Though polyphenols alone can help bone tissue regeneration, electrospun nanofiber containing polyphenols shows enhanced wound healing due to the sustained release of bioactive molecules from the scaffold

An ideal scaffold for the guided bone regeneration should be biocompatible, space-making, and permeable to fluids but acting as barriers for cells, slowly resorbable, bone-promoting, coupled with exceptional biological properties including antimicrobial ability, and commercially inexpensive [18–21]. The scaffold with these properties can be achieved in the electrospun nanofibrous membrane of the biomaterials [22–25]. During the last two decades, many researchers have shown increasing interest in the fabrication of nanofibers for bone tissue engineering applications. They develop nanofibers through multiple techniques such as electrospinning (conventional or coaxial) [26], self-assembly [27], vapor phase polymerization [28], and phase separation [29]. Among these methods, the electrospinning method is a versatile technique, which has been widely utilized to fabricate nano-fibrous scaffolds with nanosized pores and fiber diameter. The electrospun nanofibers thus prepared closely imitate extracellular matrix (ECM) with suitable mechanical property, porosity, and surface-area-to-volume ratio, which supports enhanced cell adhesion, spreading, growth, and proliferation [30]. The functional electrospun scaffold can be produced by incorporating desired biomolecules and nanoparticles into the polymeric solution (Fig. 2). The porosity and fiber diameter of electrospun fibers can be tuned by altering the parameters such as voltage, needle to collector distance, injection rate, roller speed, etc. [32]. Core-shell nanofibers are generated using a specialized coaxial electrospinning method, which uses two aligned needles that can concurrently spin two different polymer solutions [33].

The advantage of electrospun nanofibers as a drug carrier is that a greater number of drugs can be encapsulated into the scaffold compared to other forms of nanocarriers such as micelles, nanoparticles, hydrogels, etc. [29]. Further, the nanofibers can demonstrate a sustained drug release preserving the bioavailability of active drugs like polyphenols [4, 34], antibiotics [35], oligopeptides [36], medicative ingredients [37], and growth factors [38]. Various drug loading strategies lead to different kinds of interaction between drugs and nanofibers, observing different drug-releasing kinetics [39]. A curcumin-loaded PCL/gum tragacanth electrospun nanofiber was demonstrated to improve the bioavailability of curcumin, heal the wound faster, and enhance fibroblast proliferation and collagen deposition [40]. A core-shell electrospun nanofiber of PVA and PLGA loaded with naringin and metronidazole improved nanofibers’ antibacterial action, cell mobility, proliferation, and mineralization in dental application [41]. A biodegradable electrospun scaffold incorporated with transforming growth factor β-3 improved stiffness of the nanofiber and modulated chondrogenesis, and increased collagen I protein expression [42].

**Fig. 3:**
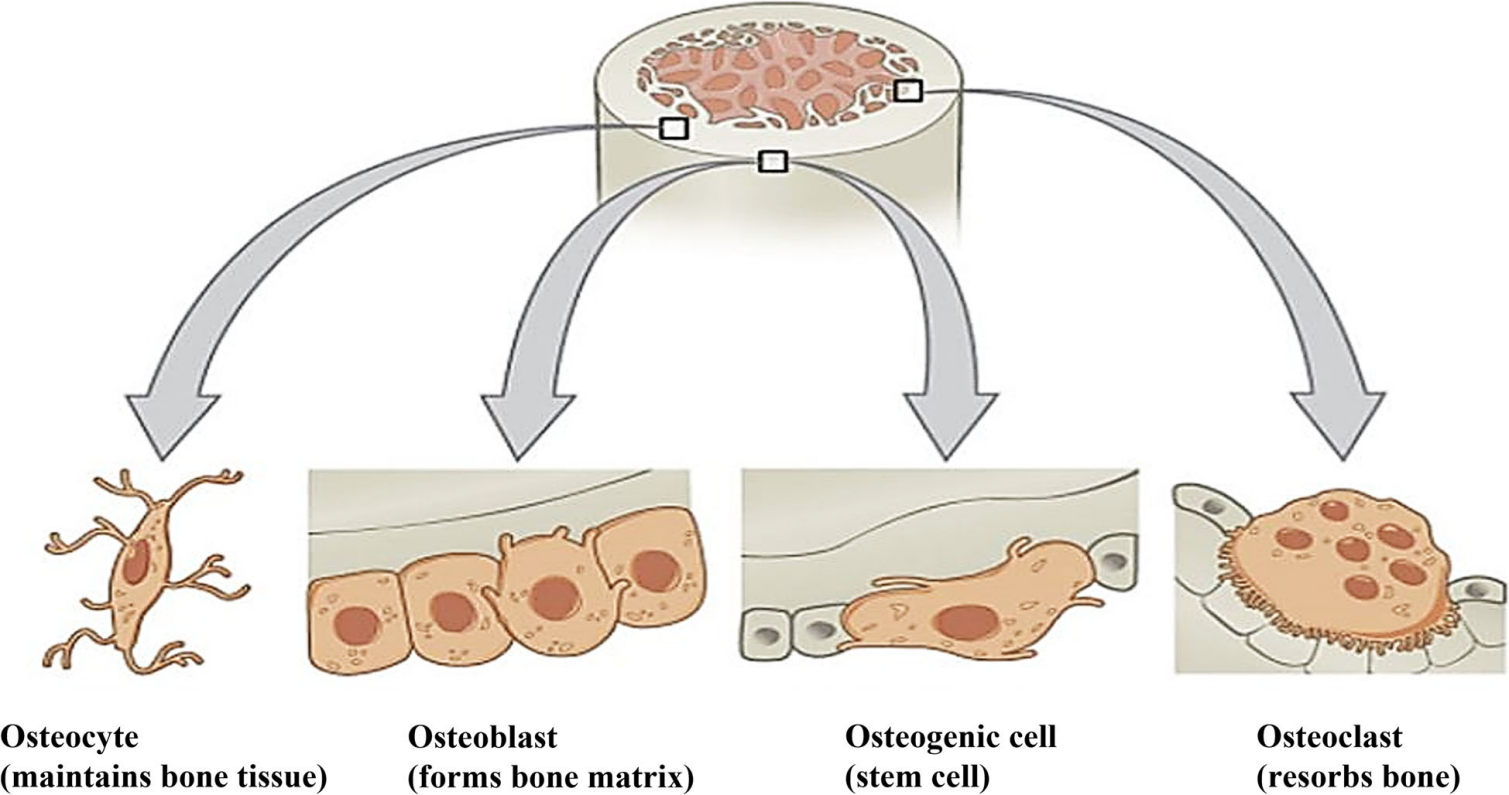
Classification of bone tissue cells. Osteogenic cells, osteocytes, osteoclasts, and osteoblast are the primary bone cells involved in bone remodeling and formation [48]

Bone is a prominent exoskeletal framework safeguarding the vital organs inside the body. The complex cellular architecture of the bone comprised 35% of organic and 65% of inorganic materials and can be classified into micro and nanocomposite tissues [43–47]. A series of biological processes, bone resorption, and bone formation make up the skeletal system, in which four major cells are involved in maintaining multiple extracellular and intracellular signaling networks (Fig. 3). Among the cells, osteoclasts and osteoblasts are responsible for bone resorption and the formation of bone matrix, respectively. These cell structures can withstand the physical pressure and maintain phosphocalcic homeostasis [49, 50]. Another cell type formed from the maturate phase of osteoblast is the osteocyte, which acts as a sensor for the endocrine responses [51]. The bone resorption and formation could be integrated using the bone cell lining, called osteogenic cells.

Bone tissue regeneration is the critical process to maintain the bone mass by repairing and regeneration. For years, 25% of trabecular bone and 3% of cortical bone have been removed and replaced through the bone regeneration process in human beings [52]. Inflammation, renewal, and bone remodeling are the three interconnecting phases involved in the bone tissue regeneration process. The inflammatory phase begins within 24 h of bone fracture or damage and continues up to a week. The blood flows into the damaged site leading to coagulation and inflammation as the immediate response after the fracture [53]. At this juncture, a series of complex signals such as proinflammatory signals and growth factors are released in a spatially controlled way [54]. Several inflammatory mediators, including interleukin-1 (IL-1, IL-6, IL-11, and IL-18) and tumor necrosis factor-α, are significantly elevated, leading to angiogenesis through inflammatory cells [55]. The platelets are activated by the damaged blood vessels to release transforming growth factor-β1 (TGF-β1) and platelet-derived growth factor [56]. Bone morphogenetic proteins are expressed by the osteoprogenitor cells at the fracture site. Inflammatory mediators along with these factors facilitate the proliferation and differentiation of mesenchymal stem cells. Stem cells differentiate into osteoblasts at the periphery of the fracture site during the renewal phase. Intramembranous ossification takes place to develop bone formation after 7 to 10 days of the bone defect. Chondrogenesis occurs at the bulk of the injured tissue, which is mechanically less stable. Endochondral bone and cartilaginous callus formation are initiated through several molecular signaling pathways, and subsequently, the calcified cartilage is replaced with woven bone [57]. In the remodeling phase, the osteoblasts with the restorative ability and the osteoclasts with resorptive ability substitute the already formed bone. Firming up of the fractured callus with a faster healing rate is observed, controlled by the proinflammatory signals and growth hormones. Within some weeks of fracture, the mechanical strength and structure are reinstated, while the molecular and cellular signaling proceeding could take up many years to restore. In a human hip fracture, the bone metabolism controlling hormone’s level remains spiked up for over a year [58]. The present review elaborates on the role of polyphenol in bone tissue engineering and their sustained activity of in-loaded electrospun nanofibers. Further, possible challenges and future directions have been discussed in this field.

## Contribution of polyphenols in bone tissue regeneration

Several in vitro and in vivo studies have been reported by many researchers to study the potential role of polyphenols in bone-related cells and bone defect models of experimental animals, respectively [59–66]. We have compiled, in this section, the source of availability, experimental parameters, and salient outcomes of polyphenols in bone tissue regeneration (Table 1).

**Table 1:** A list of polyphenols and their in vitro and in vivo experimental outcomes in bone tissue engineering

Polyphenols	Source of Availability	In Vitro/ In Vivo Biological Source	Experimental Parameters	Salient Outcomes	References
Curcumin	Beijing Solarbio Science & Technology, China	In vitro: Isolated bone marrow mesenchymal stem cells (BMSCs) from 5 to 6-week male BALB/c mice (15–21 g bw) Mouse embryonic fibroblasts (MEFs) isolated from pregnant C57/BL female mice (23–26 g bw) at 13 days of post-coitum	1) OM group: Cells in osteogenic medium 2) CR group: Cells in osteogenic medium containing 15 μM curcumin 3) ATRA group: Cells in osteogenic medium containing 1 μM all-trans retinoic acid	CR group showed an increase in the osteogenic differentiation capacity of BMSCs compared to OM and ATRA groups, as identified by the mineralization assay and RT-PCR analysis of bone markers and OCN expression. CR group augmented the osteogenic differentiation of MEFs, reprogrammed with the osteogenic factor hLMP-3. Further, it significantly increased the expression of the bone markers Runx2, BMP, and osterix at 1, 2, and 3 weeks of post-transduction.	Ahmed et al. (2019) [59]
Curcumin	Sigma-Aldrich, Germany	In vivo: Male Wistar albino rats (170–210 g bw); *n* = 10 curcumin group and *n* = 6 control group; transverse femur shaft fracture model	Control and curcumin groups (histological, biomechanical, and radiological assessment); 14 and 28 days; 200 mg/ kg oral dose in saline	The curcumin group showed no significant difference in histological, biomechanical, and radiological treatment on 14 days. No significant difference between control and curcumin-treated groups was observed on 28 days.	Safali et al. (2019) [60]
Green tea extract (GTE)	GTE Sunphenon 90LB, Taiyo International, Germany	In vitro: Primary human osteoblasts isolated from the femur heads of patients undergoing total hip replacement; 2.0 × 10^4^ cells/cm^2^	1) Control: Unstimulated cells 2) Cells stimulated six times with/without 50 μM H_2_O_2_ and 0.01, 0.1, and 1 μg/ml of GTE	Low doses of GTE improved mineralization in stimulated osteoblasts with H_2_O_2_ over 21 days. The combined effects of GTE and H_2_O_2_ led to a higher level of gene expression (osteocalcin and collagen1α1) during osteoblasts differentiation. High doses of GTE protected osteoblasts against oxidative stress by reducing intracellular free radicals and LDH leakage.	Vester et al. (2014) [61]
Green tea polyphenols (GTP)	Shili Natural Product Company, China (purity > 80%)	In vivo: Virgin 14-month-old female F344 × BFN1/NIA rats; *n* = 10/group; postmenopausal bone loss model	1) Baseline group: No surgical treatment 2) Estrogen adequate sham group (SH): SH control, SH-L (sham+ 0.1% GTP (w/v) in drinking water), and SH-H (SH + 0.5% GTP) 3) Estrogen deficient OVX group: OVX ovariectomy control, OVX-L (OVX + 0.1%), and OVX-H (OVX + 0.5%)	OVX group showed a dose-dependent increase in periosteal parameters such as mineralized bone surface and bone formation rate. However, the OVX-H group demonstrated a significant difference (*p* < 0.05) compared to other OVX groups, SH groups, and baseline group.	Shen et al. (2009) [62]
Pomace polyphenolic extract adsorbed Synergoss Red	Pomace extract: Croatina grape, Alemat, Italy Synergoss Red: Synthesized from HA, β-TCP powders, and poly (vinyl alcohol)	In vitro: Human osteoblast-like SAOS2 cells; 8.5 × 10^4^ cells/ml	0.2 g /well; 3, 5, and 7 days	The compound improved early-stage bone matrix deposition and downregulated inflammation. Further, it regulated osteoclastogenesis by the action of anti-inflammatory and antioxidant properties.	Iviglia et al. (2021) [63]
Naringin	Sigma-Aldrich, USA (purity > 95%)	In vitro: BMSCs isolated from lateral tibial tubercle of 4–8 weeks old New Zealand white rabbit (2.0 ± 0.5 kg bw)	0.1, 1, and 10 μM; 48 h 1 μM; 3, 7, 14, and 21 days	Naringin stimulated BMSCs differentiation into osteoblasts via the upregulation of miR-20a and the downregulation of PPARγ, which was significant compared to control. 1 μM of naringin significantly increased ALP expression after 3 days and showed a higher OC and Col I expression level in 21 days.	Fan et al. (2015) [64]
Apigenin	Institute of Traditional Chinese Medicine, Nanjing, China	Human fetal bone marrow-derived from the stem cells (hMSCs), Prince of Wales Hospital	Control: Osteogenic induced medium (OIM) OIM + apigenin: 0.1,1, and 5 μM; 3, 7, and 14 days.	Apigenin promoted the osteogenesis of hMSCs by stimulating JNK and p38 MAPK signaling pathways. The effect of apigenin on mRNA expression (Runx2 and OPN) in hMSCs was significantly more significant than control on 7 days (*p* < 0.01).	Zhang et al. (2015) [65]
Icariin	Tauto Biotech, Shanghai, China	In vivo: 1) 8-week-old male C57BL/6 N mice (20–25 g bw), Oriental Kobo, Japan; *n* = 5; calvarial defect model 2) 14-week-old male mice (28–33 g bw); n = 5; senescence-accelerated mouse (SAM) model	Control group: Calcium phosphate cement (CPC) tablet alone, Icariin-CPC group: CPC containing 1 mg of icariin; 4 and 6 weeks. SAM P1-control, SAMP1-icariin, SAM P6-control, and SAM P6-icariin; intraperitoneal injection; 0.2 mg/kg/day for 6 weeks.	Icariin-CPC group improved angiogenesis and accelerated bone tissue regeneration after transplantation (*p* < 0.05 compared to the control group). Among the groups, SAM P6-icariin treated mice significantly increased the trabecular bone thickness and showed a higher new bone formation rate than the control group.	Zhao et al. (2010) [66]

**Fig. 4:**
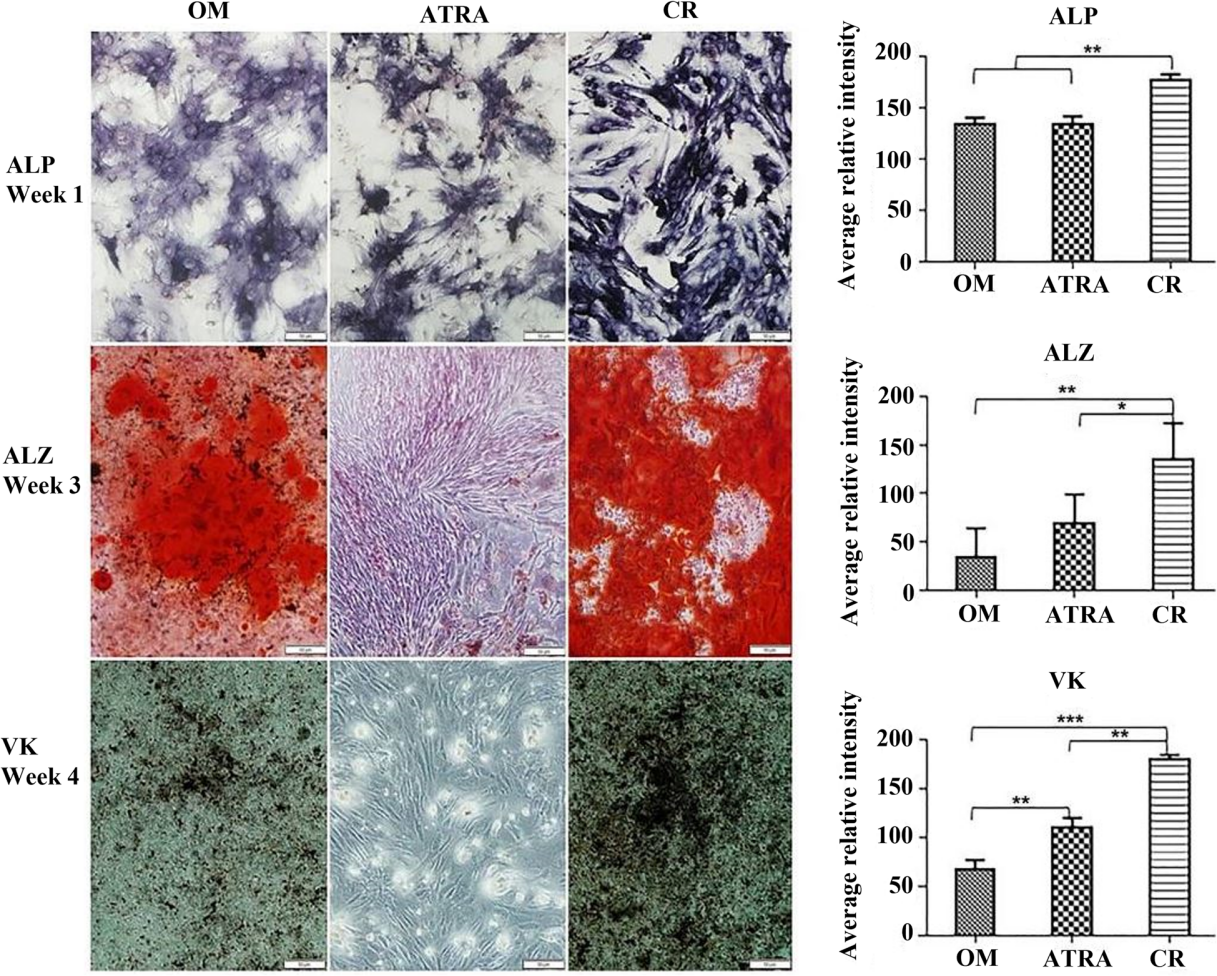
In vitro mineralization assay of bone marrow mesenchymal stem cells (BMSCs) during osteogenic differentiation following treatment with curcumin (CR) and all-trans-retinoic acid (ATRA) groups. BMSCs in the osteogenic medium were indicated by the OM group. Alkaline phosphatase (ALP), Alizarin red (ALZ), and von Kossa (VK) staining results were obtained after 1, 3, and 4 weeks of post-induction, respectively. Quantification of staining intensity was performed with ImageJ software. The level of calcium deposition reflects the extent of mineralization, which was higher in the CR group than in the OM and ATRA groups. ATRA group did not show any symptoms of mineralization. The data were represented as the mean ± standard deviation (*n* = 3). The statistical significance was defined using a one-way analysis of variance, followed by Tukey’s post hoc test. *p < 0.05, **p < 0.01 and, ***p < 0.001 [59]

Curcumin, extracted from the rhizome of *Curcuma longa*, has become a subject matter as a potential therapeutic agent in the orthopedic field [67]. Curcumin supplementation has been proven to be efficient in preventing and managing osteopenia and has been reported to have beneficial effects on fat metabolism and bone health [68]. The potential mechanisms of curcumin include inhibition of nuclear factor NF-κB, RANKL, inflammatory cytokine synthesis, and the generation of reactive oxygen species and nitric oxide [69, 70]. Ahmed et al. studied the effect of curcumin (CR group) on osteogenic differentiation of bone marrow mesenchymal stem cells (BMSCs) and mouse embryonic fibroblasts (MEFs) compared with all-trans-retinoic acid (ATRA group) and osteogenic medium only as control (OM group). Curcumin stimulated osteogenic differentiation at the cellular and molecular level and increased the expression of osteogenic differentiation markers such as Runx2, osterix, and BMP2. The positive effect of curcumin showed a strong ALP staining intensity, higher mineralization, and upregulation of osteo specific bone markers, confirming an improved osteogenic differentiation of BMSCs compared with ATRA and control (Fig. 4). Moreover, it enhanced the osteogenic differentiation in MEFs reprogrammed with the osteogenic factor hLMP 3, participating in the regulation of bone remodeling [59]. Safali et al. examined the effects of curcumin on bone healing using a total rat femur fracture injury model. Unexpectedly, they found that curcumin had no effect on fracture healing based on biomechanical, radiological, and histological evaluations on 14 and 28 days of investigation. However, they suggested that curcumin’s impact may be more noticeable in long-term follow-up investigations because of its potential positive effects, such as activation of cell migration and autophagy during the remodeling phase [60].

Vester et al. examined the dose- and time-dependent effect of green tea extracts (GTE) in human osteoblasts, isolated from femoral heads of patients undergoing total hip replacement. They performed RT-PCR to access the combined effects of GTE (0.01, 0.1, and 1 μg/ml) and H_2_O_2_ (50 μM) on the osteogenic genes and found significant expression of bone-related genes such as osteocalcin and collagen1α1 during osteoblast differentiation. They reported that GTE, at all the concentrations studied, enhanced the mineralized matrix development despite H_2_O_2_ treatment. Further, GTE significantly reduced oxidative stress improving cell viability, suggesting that dietary supplementation of GTE could reduce inflammatory reactions in bone-related diseases such as osteoporosis. Osteoporosis is characterized by structural deterioration of bone tissue and low bone mass causing bone fragility [61]. Shen et al. investigated whether green tea polyphenol (GTP) has the potential to restore bone microstructure in both estrogen adequate (sham group) and estrogen-deficient (OVX group) middle-aged female rats. According to HPLC-ECD and HPLC-UV analyses, GTP (1000 mg) contained a mixture of epigallocatechin gallate (480 mg), epicatechin gallate (160 mg), epicatechin (60 mg), epigallocatechin (103 mg), and catechin (30 mg). The analyses of dual-energy X-ray absorptiometry, micro-computed tomography, and histomorphometry revealed that GTP supplementation increased trabecular thickness, volume, and number and periosteal bone formation rate of tibia shaft, cortical thickness, and femur area. Meanwhile, GTP decreased bone erosion of proximal tibia, trabecular separation, and endocortical bone erosion of the tibia shaft. OVX rat groups demonstrated a dose-dependent increase with GTP in the parameters such as mineralized bone surface and bone formation rate. In contrast, SH rat groups with GTP did not show any significant difference [62].

A new ceramic granulated biomaterial (Synergoss Red, SR) was functionalized with red grape pomace extract containing polyphenolic mixture to study its regeneration effect on periodontal tissues [63]. The primary polyphenols present in pomace extract, including quercetin [71], kaempferol [72], and catechins [73], were shown to direct osteogenic differentiation in different mesenchymal stem cell types. The bone filler, SR, was synthesized from the mixture of 47 wt% of hydroxyapatite (HA) and tricalcium phosphate (β-TCP) powders with the binding agent, 3 wt% of poly(vinyl alcohol). A 0.2 g of Synergoss Red was capable of adsorbing around 0.951 mg of polyphenols. Several studies have confirmed that polyphenols exhibit antioxidant properties naturally and involve many bone regeneration mechanisms [74, 75]. In the present study, the compound showed free radical inhibition by 72.8%, characterized by a DPPH assay. Polyphenols in Synergoss Red significantly reduced the level of iNOS expression (*p* < 0.0001) compared to control (bone filler alone). The anti-inflammatory and antioxidant properties of polyphenols in pomace extract exerted a protective role in bone loss by reducing osteoclastogenesis and enhancing osteoblastogenesis.

Naringin, a dihydrotestosterone flavonoid compound, has been reported to improve bone density, inhibit bone loss, and augment biomechanical anti compression performance [76]. Fan et al. studied the osteogenic differentiation ability of naringin in BMSCs collected from the lateral tibial tubercle of the white rabbit. The treatment of BMSCs with 0.1, 1, and 10 μM naringin for 48 h significantly increased the mRNA expression levels of OC, ALP, and Col I, compared to control (without additive). The western blot and RT-PCR analyses showed a decreased PPARγ protein expression and an increased miR-20a marker expression in BMSCs when the cells were treated with 1 μM of naringin for 21 days. The results suggested that naringin, as a potential drug, may promote BMSCs’ differentiation into the osteoblasts during osteoporosis treatment [64]. Apigenin (4′,5,7-trihydroxyflavone), a member of the flavone family of flavonoid compounds, was reported to possess remarkable anti-carcinogenic, antioxidant, and estrogenic properties [77]. Zhang et al. studied the transducing ability of apigenin in hMSCs into osteoblasts and reported that apigenin significantly increased activity of ALP and the mineralized nodule formation in a dose-dependent manner [65]. The cells treated with 5 μM of apigenin significantly increased Runx2 and OSX protein expression through JNK and p38 MAPK pathways, which play a pivotal role in regulating the osteogenic differentiation of MSCs [78].

The research group of Zhao et al. evaluated the osteogenic effect of icariin through in vitro and in vivo biological characterizations [66]. Icariin, a flavonoid glycoside isolated from the herb of Epimedium pubescens, has been reported to have potential therapeutic effects on a rat model of osteoporosis induced by ovariectomy [79, 80]. Preosteoblast MC3T3-E1 and fibroblast NIH3T3 cells were used for the in vitro osteogenesis analysis in work. MC3T3-E1 cells treated with 10^− 5^ M of icariin exhibited a significant increase in ALP activity, Runx2, bone sialoprotein (BSP), and osteocalcin (OCN) expression at day 3. In contrast, icariin-treated fibroblast cell line NIH3T3 had not shown remarkable ALP and protein expression. Two types of animal models, viz. calvarial defect model and senescence-accelerated mouse models, were investigated to study icariin’s bone regeneration ability in vivo. In the calvarial defect model, eight-week-old male C57BL/6NJ mice were transplanted with icariin-calcium phosphate cement (CPC) tablets or CPC tablets only (control) to evaluate bone tissue regeneration after 4 and 6 weeks. The icariin-CPC group demonstrated significant new bone formation and new bone thickness at 4 weeks and 6 weeks, respectively, compared to the control group. The senescence models (SAM P1 and SAM P6) revealed that icariin injected mice could enhance bone formation in vivo. Overall, the results suggested that icariin could act as a strong candidate for an osteogenic compound in bone tissue engineering applications.

## Advantages of polyphenol-loaded electrospun nanofibers

Biocompatible and naturally available biopolymers and synthetic polymers have been widely used to prepare electrospun nanofibrous mats for tissue engineering applications [81–83]. The current situation demands the fabrication of highly bioactive scaffolds with superior biocompatibility, mechanical properties, and remodeling potential to repair the damaged tissues. The same can be achieved by either surface functionalization or incorporation of bioactive materials in the nanofiber membrane. The nanofiber scaffold’s primary goal is to provide an appropriate microenvironment for bone tissue to restore and facilitate the bone tissue regeneration process [84]. Ideally, the fabrication of polyphenol-loaded electrospun nanofibers scaffolds has some advantages in bone tissue regeneration applications. They exert anti-inflammatory and antioxidant activity, improve bioavailability, and release the polyphenols at a sustained level in the cell differentiation site. They provide an active shield against infection, minimize toxicity to other tissues, and enhance the bone remodeling process via calcium deposition and activation of several bone-specific proteins [85]. The incorporated biomolecules into the scaffold can interact with the biomaterial’s surface through various physical and chemical forces, including hydrogen bonding, hydrophobic interaction, and Van der Waals force [86]. It was reported that the ion complexation property of bioactive molecules could cause protein deactivation and denaturation [87, 88]. The surface-functionalization of nanofibers with bioactive molecules via non-covalent immobilization techniques protects the nature of the bioactive molecules and the structure of biomaterials [89, 90]. Further, the bioactive molecules improve hydrophilicity and surface charge of the nanofiber’s surface, establishing a favorable milieu, enhancing the protein adsorption on its surface [91, 92]. Henceforth, the fabrication of electrospun nanofibers using biocompatible polymer or specific polyphenols expands their mechanical, biological, and functional properties, leading to cell attachment, cell migration, and cell proliferation [93]. A list of polyphenols incorporated electrospun nanofibers and their application in bone tissue regeneration has been provided in Table 2.

**Table 2:** The preparation method of polyphenol-loaded electrospun nanofiber, nanofiber diameter distribution, and their contribution to bone tissue engineering are listed

Polyphenol Additives	Polymeric Composite with Additives and their Labels	Electrospinning Method and the Nanofiber Diameter Distribution	In Vitro / In Vivo Biological Source	Salient Outcomes	References
Curcumin	PCL-curcumin (CU0, CU1, and CU5)	Conventional method/ CU0: 840 ± 130 nm CU1: 827 ± 129 nm CU5: 680 ± 110 nm	In vitro: MC3T3-E1 mouse pre-osteoblasts; 1, 5 and 10 days	CU1 nanofibers showed significant osteogenesis leading to mineralization compared to CU0 and CU5 nanofibers.	Jain et al. (2016) [94]
Curcumin	4-arm PCL-(Zn-curcumin)/ PVA-CMCh-GO (N1, N2, N3, N4, and N5)	Coaxial method/ N1: 205 ± 92 nm N2: 186 ± 78 nm N3: 174 ± 56 nm N4: 153 ± 31 nm N5: 156 ± 34 nm	In vitro: MG-63 human osteoblasts; 7 and 14 days.	The experimental nanofiber (N4) showed an increased ALP activity, enhanced matrix mineralization, and reduced post-operative infection.	Sedghi et al. (2018) [95]
Catechin (Cat)	PCL-Cat	Conventional method/ PCL: 200 ± 150 nm PCL-Cat: 200 ± 150 nm	In vivo: critical-sized calvarial bone defect mouse model; 4 mm defect size; 8 weeks Control (no treat), PCL scaffold, PCL-Cat, PCL-hADSC, and PCL-Cat-hADSC groups	PCL-Cat-hADSC demonstrated a high bone coverage and bone volume than other groups on 8 weeks of post-transplantation (*p* < 0.01 vs. control; *p* < 0.05 vs. PCL)	Lee et al. (2017) [96]
Polyhedral oligomeric silsesquioxane-epigallocatechin gallate (POSS-EGCG)	Poly(vinylidene fluoride)-POSS-EGCG (PVDF, PE02, PE04, and PE06)	Conventional method/ PVDF: 1033 ± 270 nm PE02: 971 ± 262 nm PE04: 936 ± 223 nm PE06: 1094 ± 394 nm	MC3T3-E1 osteoblasts; 3, 5, 7, and 14 days; 1 × 10^4^ cells	POSS-EGCG conjugation improved bioactivity of PVDF nanofiber; PE06 showed maximum ALP activity and improved bone mineralization (*p* < 0.05 vs. PVDF).	Jeong et al. (2019) [97]
Zinc quercetin-phenanthroline (Zn + Q(PHt))	PCL-gelatin- (Zn + Q(PHt))	Conventional method/ PCL-gelatin: 260–500 nm PCL-gelatin-(Zn + Q(PHt)): 250–600 nm	In vitro: MG-63 osteoblast-like cells; 3 and 7 days	PCL-gelatin-(Zn + Q(PHt)) scaffold showed more relative ALP activity than PCL-gelatin on 3 and 7 days of post-treatment; Runx2 and type 1 collagen mRNAs expression were also found more significant in PCL-gelatin-(Zn + Q(PHt)) scaffold.	Preeth et al. (2021) [98]
Resveratrol (RSV)	PCL-RSV and PLA-RSV	Conventional method/ PCL-RSV: 0.97 ± 0.45 μm PLA-RSV: 0.45–1.20 μm	In vitro: STRO-1 positive stem cells (STRO-1^+^ cells); 1, 3, 7, 14, and 21 days	Both materials exhibited the same level of osteoinductive capacity; Only PLA-RSV induced expression of osteoblasts inhibiting osteoclast differentiation.	Riccitiello et al. (2018) [99]
Icariin (ICA)	PG: PCL-gelatin nanofiber without drug PGM: nanofiber with MOX PGI: nanofiber with ICA PGMI: nanofiber with MOX-ICA	Coaxial method/ PG: 0.4–0.8 μm PGM: 0.4–0.8 μm PGI: 0.7–1 μm PGMI: 0.7–1 μm	In vitro: MC3T3-E1 cells; 7, 14, and 21 days In vivo: New Zealand White rabbits; 2.5 kg bw; 3 groups; 1, 2, and 3 months	PGI promoted a significant ALP secretion among all the fiber membranes, whereas PGMI demonstrated a higher expression of OCN and COL I. PGMI group displayed a high quality of bone formation compared to untreated and PG groups at 3 months of post-surgery.	Gong et al. (2019) [100]
Icariin	PCL-gelatin-icariin (PGI0, PGI0.005, PGI0.01, PGI0.05, PGI0.1, and PGI0.5)	Conventional method/ PGI0: 0.26 ± 0.06 μm PGI0.005: 0.19 ± 0.05 μm PGI0.01: 0.17 ± 0.04 μm PGI0.05: 0.16 ± 0.05 μm PGI0.1: 0.17 ± 0.04 μm PGI0.5: 0.16 ± 0.04 μm	In vitro: MC3T3-E1 cells; 14 and 21 days	PGI0.05 efficiently enhanced the expression of ALP, OCN, COL 1, and calcium deposition compared to other scaffolds.	Gong et al. (2018) [101]

Jain et al. prepared curcumin-loaded PCL electrospun nanofibers (CU1 and CU5) to investigate the influence of curcumin drug release from the scaffold on osteogenesis and compare the results with PCL scaffold without drugs (CU0). It was found that both fiber mats released around 18% of the drugs on day 3. However, CU1 and CU5 showed different drug releases of 42 and 50%, respectively, on day 6 of the investigation. The in vitro results using MC3T3-E1 mouse pre-osteoblasts demonstrated that ALP activity of the scaffolds was found in the order of CU1 > CU0 > CU5. The optimized concentration of curcumin and sustained drug release from CU1 helped increase osteogenic expression compared to CU5, which had a high drug loading content [94]. Sedghi et al. developed bioactive molecule-loaded coaxial electrospun nanofibrous scaffolds with anti-infective properties to prove effective bone tissue regeneration. The bioactive complex was composed of zinc-curcumin (Zn-CUR) and graphene oxide. The developed core-shell nanofiber membrane comprised a blend of polyvinyl alcohol and carboxymethyl chitosan (PVA/CMCh) in the shell and 4armPCL/Zn-CUR in the core part. Cellular morphology and MTT (3-(4,5-Dimethylthiazol-2-yl)-2,5-Diphenyltetrazolium bromide) assay showed that Zn-CUR-containing scaffolds substantially supported cellular adhesion, spreading, and proliferation compared to drug-free scaffolds. Moreover, the Zn-CUR complex in the scaffolds increased ALP activity and matrix mineralization and reduced postoperative infection with an excellent antibacterial activity as the metal-organic complex improves the bioavailability of curcumin. Further, complex localization into the core part of the core-shell nanofiber leads to its controlled release, enhancing its therapeutic efficiency [95].

**Fig. 5:**
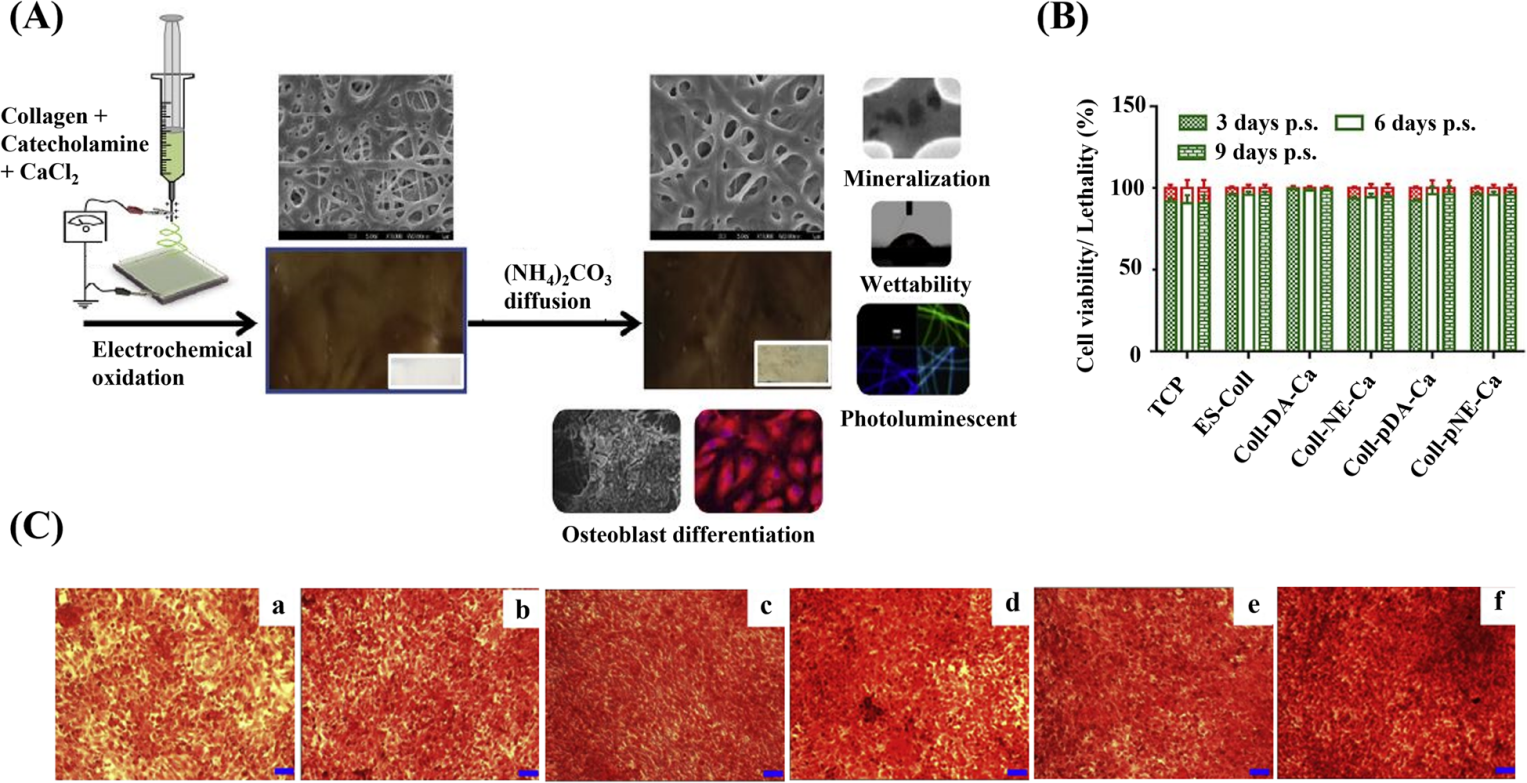
Analyses of in vitro cell viability and calcium deposition of catecholamine containing collagen nanofiber. **A** Electrospun nanofibrous mat was prepared from the composite of collagen (8% w/v), dopamine (10% w/w of collagen), and 20 mM CaCl2 in 90% HFIP. The brown coloration in the mats is due to the formation of polydopamine by electrochemical oxidation. Intensified brown color in mat and precipitation of CaCO3 occurred by the subsequent exposure of the mat to (NH4)2CO3 vapors. The nanofibrous mat exhibited excellent mechanical properties, surface wettability, fluorescence, and osteoblast cell proliferation and differentiation. **B** Human fetal osteoblastic cell line (hFob) viability was quantified from live/dead cell ratio cultured on various collagen scaffolds and tissue culture plate (TCP) (Mean ± SD, *n* = 3). **C** Calcium deposition on various collagen mats by ARS (Alizarin Red S) staining with scale bar = 50 μm. (a) TCP, (b) Pristine collagen mats (ES-Coll), (c) As-spun collagen mats with DA and 20 mM Ca2+ (Coll-DA-Ca), (d) As-spun collagen mats with NE and 20 mM Ca2+ (Coll-NE-Ca), (e) Collagen mats after (NH4)2CO3 exposure (Coll-pDA-Ca), and (f) Coll-pNE-Ca [102]

**Fig. 6:**
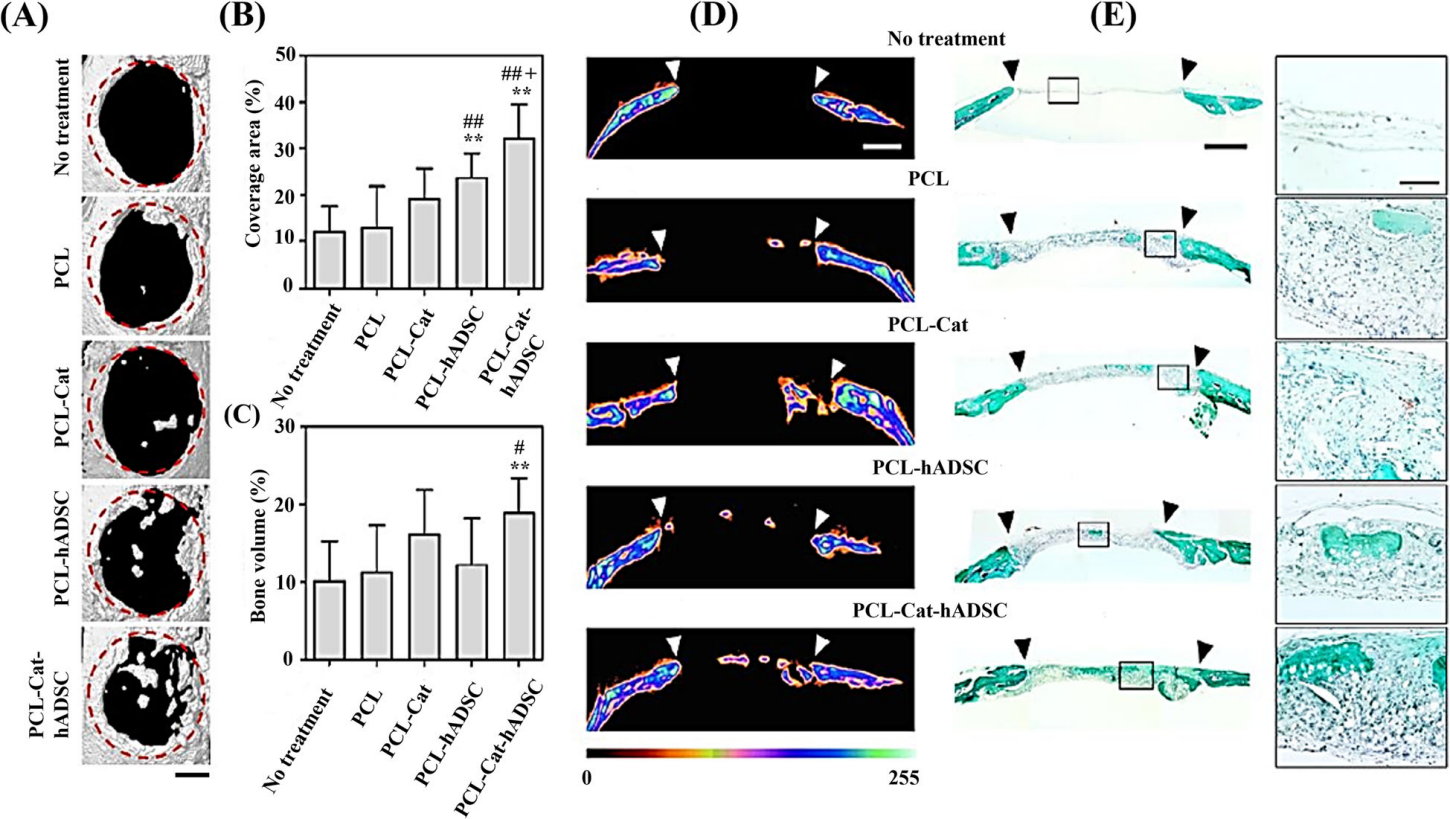
In vivo bone regeneration of polycaprolactone (PCL), catechin-loaded PCL (PCL-Cat), hADSC transplanted PCL (PCL-hADSC), and Cat-hADSC transplanted PCL (PCL-Cat-hADSC) scaffolds in critical-sized calvarial bone defect mouse model. **A** Micro-CT images of the defect site have been seen with a scale bar of 1 mm on 8 weeks of post-transplantation. Quantification of bone coverage area (%) and bone volume (%) has been shown in (**B**) and (**C**), respectively (*n* = 10, **p < 0.01 vs. no treatment group; #*p* < 0.05 and ##*p* < 0.01 vs. PCL group; +*p* < 0.05 vs. PCL-Cat group). **D** Colorized mineral map of the cross-sectioned micro-CT images with scale bar 1 μm. The defect region is indicated with white arrowheads. (**E**) Goldner’s Trichrome staining of each group (left, 1 mm) with their expanded images (right, 100 μm). Black arrowheads indicate the defect region [96]

Dhand et al. fabricated catecholamine contained collagen nanofiber with excellent mechanical property without interfering with hydrophilicity of the nanofiber surface [102]. The in vitro cell viability and calcium deposition analysis confirmed that the highly biocompatible catecholamine contained composite nanofiber enhanced calcium mineralization (Fig. 5). Lee et al. prepared catechin coated functional PCL nanofibrous scaffolds with antioxidative property and calcium-binding ability to achieve an enhanced osteogenic differentiation of human adipose-derived stem cells (hADSCs). The scaffold was reported to significantly promote in vivo bone formation in a critical-sized calvarial bone defect. The scaffolds were divided into five different groups, no treatment, PCL scaffold (PCL), catechin coated PCL scaffold (PCL-Cat), PCL with hADSCs (PCL-hADSC), and catechin-coated PCL scaffold with hADSCs (PCL-Cat-hADSC). The results of micro-CT images and histological examination demonstrated that PCL-Cat-hADSC showed an improved tissue regenerative efficacy by the influence of catechin (Fig. 6) [96].

Jeong et al. developed polyhedral oligomeric silsesquioxane-epigallocatechin gallate (POSS-EGCG) loaded poly (vinylidene fluoride) electrospun nanofiber to investigate bone tissue regeneration [97]. Epigallocatechin gallate (EGCG), a polyphenolic flavonoid derived from a variety of plants, has been reported to impede lipopolysaccharide (LPS)-stimulated osteoclastic bone resorption and reduce inflammatory bone loss in bone metabolism [103, 104]. It was found that the concentration of 6 wt% POSS-EGCG conjugates (PE06) loaded PVDF nanofibrous scaffold showed betterment in physiochemical and mechanical properties than 0, 2, and 4 wt% conjugate loaded scaffolds (PVDF, PE02, and PE04, respectively). The in vitro osteogenic and osteoblast differentiation results confirmed that PE06 exhibited a higher ALP activity and bone mineralization than other scaffolds. Our recent work demonstrated a promising PCL-gelatin-(Zn + Q(PHt)) nanofibrous scaffold to increase osteoblastogenesis, leading to bone formation. The metal-organic complex (Zn + Q(PHt)) was synthesized by refluxing a methanolic mixture of phenanthroline (PHt), quercetin, and Zn^2+^ ions. The fibers diameter distribution of PCL-gelatin-(Zn + Q(PHt)) scaffold was 250–500 nm with 72% of bioactive complex entrapped into nanofiber matrix. The in vitro and in vivo biological studies indicated that the scaffold exhibited a better osteogenic differentiation with a large amount of Runx2 and type 1 collagen mRNAs expression than the PCL-gelatin nanofiber alone [98].

Resveratrol (RSV), a natural polyphenolic compound, is present in numerous plant products, including red wine. Its biological effects are antioxidant, anticancer, anti-inflammatory, cardiovascular protection, antiaging, and bone-protective property [105, 106]. It stimulates osteoblast differentiation in a dose-dependent manner activating MAPK signaling pathway and further inhibits bone resorption by constraining RANKL-induced osteoclast differentiation [107]. RSV has been used for the alveolar socket reduction and remodeling of the dental implant after removing the tooth [108, 109]. However, the oral bioavailability of RSV is limited due to its low water solubility, poor pharmacokinetics, and instant metabolism. Hence, it is required to formulate a sound delivery system to deliver RSV at the target site [110]. Riccitiello et al. prepared RSV-loaded defect-free PCL (PCL-RSV) and poly(lactic) acid electrospun nanofibers (PLA-RSV) for the treatment of alveolar bone defect. Both PCL-RSV and PLA-RSV promoted human dental pulp stem cells (DPSCs) differentiation into osteoblast-like phenotype, triggering the expression of early (Runx2 and OSX) and late (OCN, ONN, OPN, and BSP) osteoblast differentiation markers. Though PLA-RSV contained a lower number of drugs than PCL-RSV, only the former scaffold could induce osteoblast differentiation and inhibit osteoclast differentiation, suggesting its use in preserving the post-extraction alveolar ridge volume during bone resorption and new bone formation [99].

Gong et al. fabricated an icariin-loaded core-shell electrospun membrane to imitate artificial periosteum for bone tissue regeneration. They prepared different core-shell type electrospun nanofibrous scaffolds from PCL and gelatin polymeric components and labeled them as PG (without drug), PGM (MOX loaded), PGI (icariin loaded), and PGMI (dual drug-loaded). All the prepared scaffolds showed membrane degradation up to 60–80% for over 2 months. The sustained release of icariin from the nanofiber PGI helped augment osteogenic differentiation, especially ALP expression and Ca^2+^ deposition. However, dual drug-loaded PGMI was effective in showing more expression of OCN and COL I. The histology, immunohistochemical and radiographic results demonstrated that the quality of bone formation and the quantity of bone mass was highly enhanced in the PGMI group than that of PG and untreated rabbit groups [100]. In another research work, the same researcher prepared various PCL/gelatin nanofibrous scaffolds with different concentrations of icariin viz. 0, 1.2, 2.4, 12, 24, and 120 mg/ml and labeled as PGI0, PGI0.005, PGI0.01, PGI0.05, PGI0.1, and PGI0.5, respectively. They found that PGI0.05 possessed exceptional overall performances related to bone regeneration by accelerating OCN, ALP, COL I, and calcium expression. Further, the degradation behavior and mechanical strength of PGI0.05 were also reported to fulfill the requirements of an artificial periosteum. Hence, the PGI0.05 scaffold was recommended as a potential artificial periosteum to repair large-sized bone defects [101]. The compilation of research materials demonstrates that polyphenols contribute to enhancing bone tissue regeneration as the drug molecules alone or the active components in scaffolds like electrospun nanofibers.

## Conclusion and future perspectives

This review demonstrated the in vitro and in vivo bone tissue regenerating ability of polyphenols with or without the electrospun nanofibrous scaffold. The beneficial properties of polyphenols loaded nanofibrous scaffolds are antioxidant property, biocompatibility, porosity, flexibility, tensile strength, cell proliferation, and osteogenic differentiation. However, there are some issues to be addressed in this field. (1) Though many reports are available to study polyphenols loaded incorporated electrospun nanofiber for bone tissue regeneration, the nanofibers prepared in their studies are mostly accomplished using the conventional electrospinning method. Only a few reports are available to prepare core-shell nanofibers using coaxial methods, releasing polyphenols with a desired drug delivery profile. According to the literature reports, the core-shell structured nanofiber permits the encapsulation of sensitive bioactive molecules into the core portion for better loading and controlled long-term release compared to normal nanofiber [95]. Hence, the researchers should focus on loading polyphenols into differently structured nanofibers to increase their therapeutic efficacy. (2) The researchers should conduct drug loading/release profiles using different polyphenols in the nanofibrous mats to provide comparative data. (3) The bone tissue regeneration phase, in which the polyphenols contribute more effectively, should be investigated through many systematic biological studies. (4) The selection of suitable polyphenols for scaffold fabrication is still an open question in most cases due to the non-specific regulations and theoretically indistinguishable structure-function performance. So, more works need to be carried out to identify which polyphenols category is most suitable for bone tissue engineering applications. (5) The nature and functionality of the polyphenols should be analyzed when they are released from the nanofibrous mats because the polyphenols may alter their molecular structures and dissolution depending on the external medium. We are suggesting the following comments for the future direction in this field. (1) Characterizing more in vitro and in vivo biological studies based on polyphenol-loaded electrospun nanofibrous mats prepared by the coaxial method. (2) Tracking the presence of polyphenols and determining their quantity in different bone tissue regeneration phases using appropriate sophisticated methods. (3) Analyzing the nature of polyphenols before and after loading into the scaffolds using spectral characterizations such as HPLC and NMR. (4) Performing more molecular level studies to gain insights into the cellular mechanism in which polyphenols are involved. We anticipate that researchers with interdisciplinary backgrounds will develop bone tissue regeneration by emphasizing polyphenols’ importance and suggestions.

## Data Availability

Not applicable.

## Abbreviations

GO: Graphene oxide
CMCh: Carboxymethyl chitosan
Zn-CUR: Zn-Curcumin complex
PLA: Polylactic acid
PCL: Polycaprolactone
GEL: Gelatin
CAT: Catechin
BMC: Bioactive metal complex
BMP: Bone morphogenetic protein
MOX: Moxifloxacin
BMSCs: Bone marrow mesenchymal stem cells
bw: Body weight
ALP: Alkaline phosphatase
OCN: Osteocalcin
OSX: Osterix/ Transcription factor Sp7
JNK: c-Jun N-terminal kinase
ONN: Osteonectin
OPN: Osteopontin
Runx2: Runt-related transcription factor 2
BSP: Bone sialoprotein
RANKL: Receptor activator of nuclear factor kappa-Β ligand
Col I: Collagen type 1
PPARγ: Peroxisome proliferator- activated receptor gamma
HPLC: High performance liquid chromatography
NMR: Nuclear magnetic resonance
RT-PCR: Real-time polymerase chain reaction
